# Supplementary material for: Rapid and inducible mislocalization of endogenous TDP43 in a novel human model of amyotrophic lateral sclerosis
Source: eLife. 2025 Jul 24;13:RP95062. doi: 10.7554/eLife.95062 (PMC12289307; doi:10.7554/eLife.95062)
Supplement: Figure 1—figure supplement 1—source data 1. [file elife-95062-fig1-figsupp1-data1.zip › Figure 1-Figure supplement 1 -Source data 1/Figure 1-figure supplement 1-Source data 1.pdf]

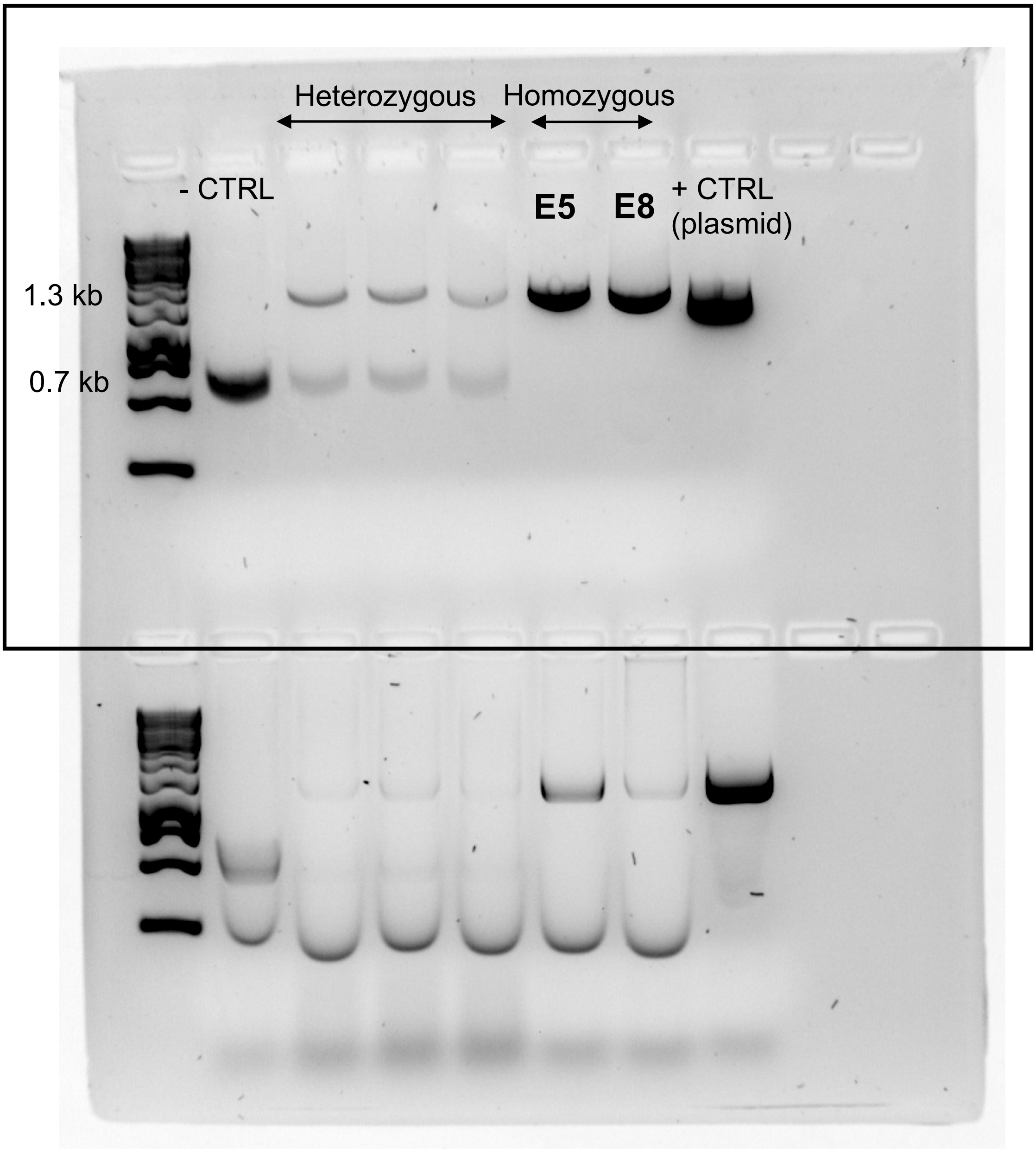

Figure 1-Figure supplement 1-Source data 1. Uncropped Gel from PCR screen for TDP43-GFP KI clones.
Confirmation of three heterozygous and two homozygous TDP43-GFP knock-in clones using PCR-gel electrophoresis. The unedited parent healthy iPSCs served as a negative control and a TDP43-GFP plasmid served as a positive control. Homozygous “E5” and “E8” clones were selected. The black box indicates blots shown in paper Figure 1–figure supplement 1A.
The panel below shows the same samples, but without Ampure purification prior to gel electrophoresis. CTRL = control.
