# Supplementary figures and images for: Rapid and inducible mislocalization of endogenous TDP43 in a novel human model of amyotrophic lateral sclerosis
Source: eLife. 2025 Jul 24;13:RP95062. doi: 10.7554/eLife.95062 (PMC12289307; doi:10.7554/eLife.95062)

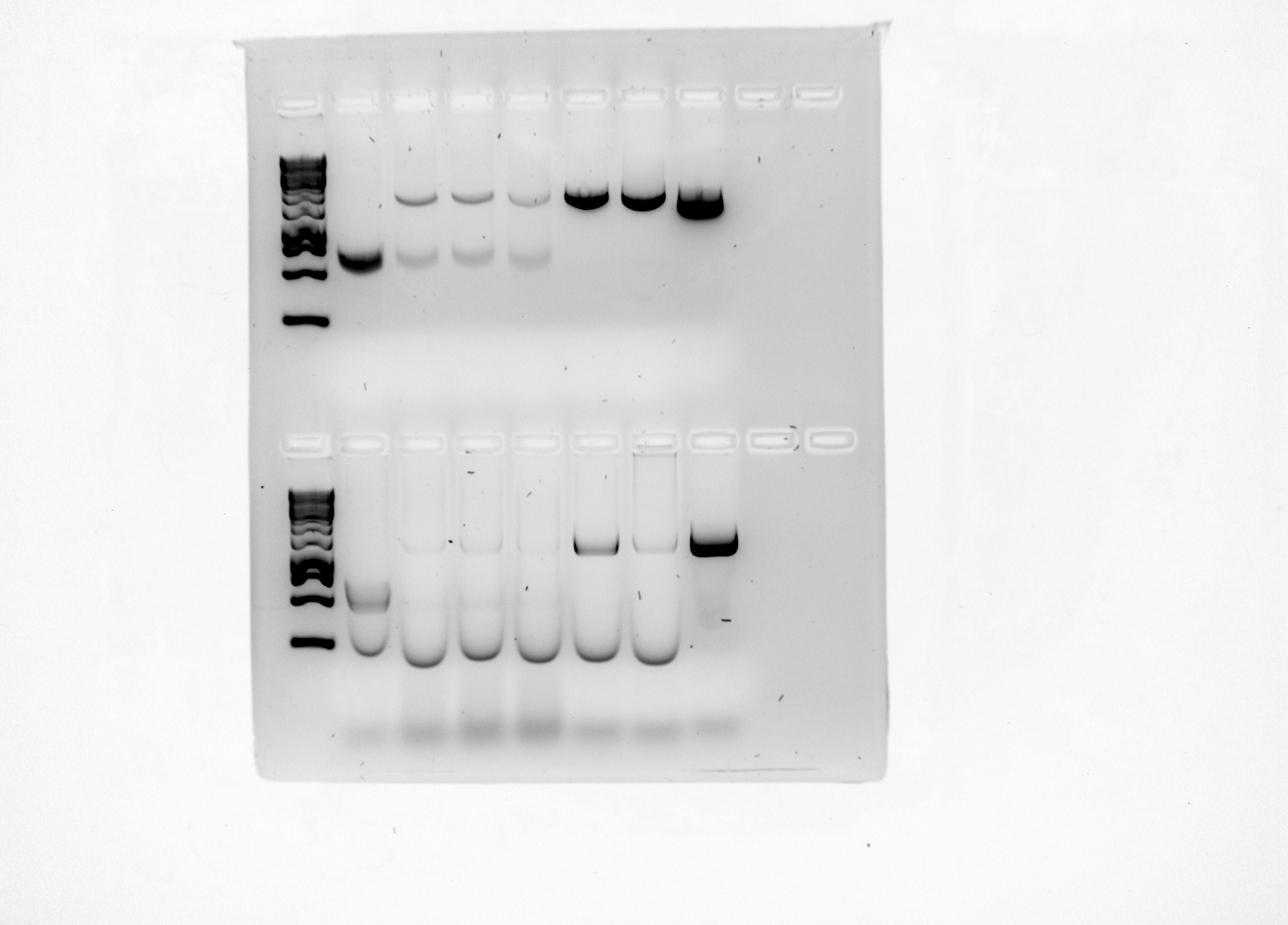

Supplement: Figure 1—figure supplement 1—source data 2. [file elife-95062-fig1-figsupp1-data2.zip › Figure 1-figure supplement 1-Source data 2/Figure 1-figure supplement 1-Source data 2.tif]

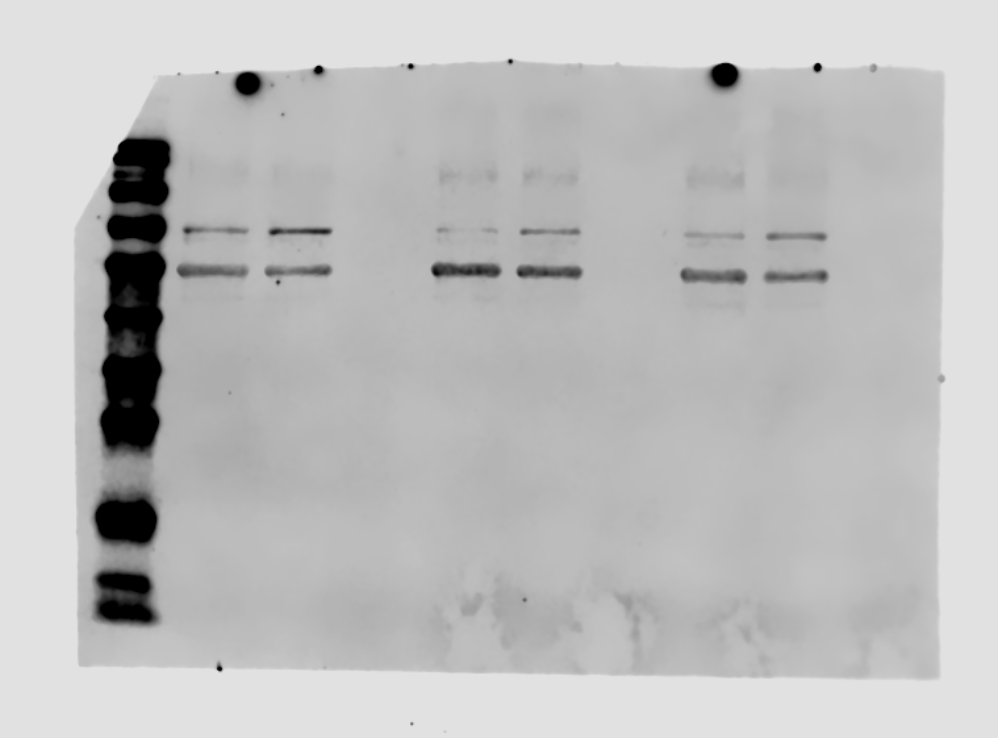

Supplement: Figure 3—source data 2. [file elife-95062-fig3-data2.zip › Figure 3-Source data 2/Figure 3-Source data 2- pTDP43.tif]

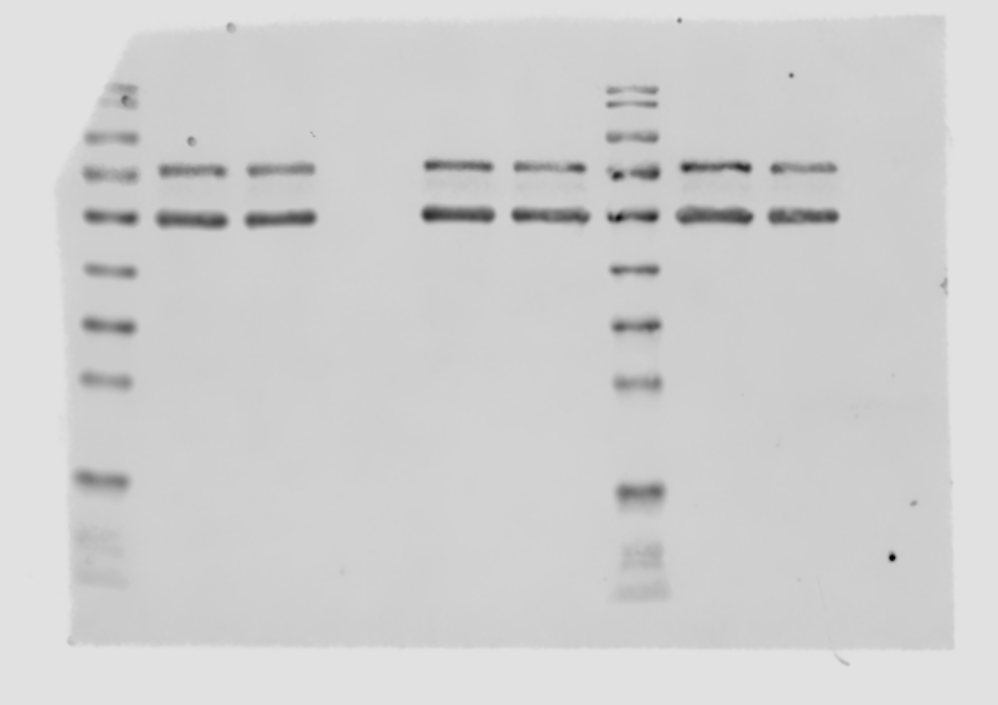

Supplement: Figure 3—source data 2. [file elife-95062-fig3-data2.zip › Figure 3-Source data 2/Figure 3-Source data 2-total TDP43.tif]
