# Supplementary material for: Rapid and inducible mislocalization of endogenous TDP43 in a novel human model of amyotrophic lateral sclerosis
Source: eLife. 2025 Jul 24;13:RP95062. doi: 10.7554/eLife.95062 (PMC12289307; doi:10.7554/eLife.95062)
Supplement: Figure 3—source data 1. [file elife-95062-fig3-data1.zip › Figure 3-Source data 1/Figure 3-Souce data 1.pdf]

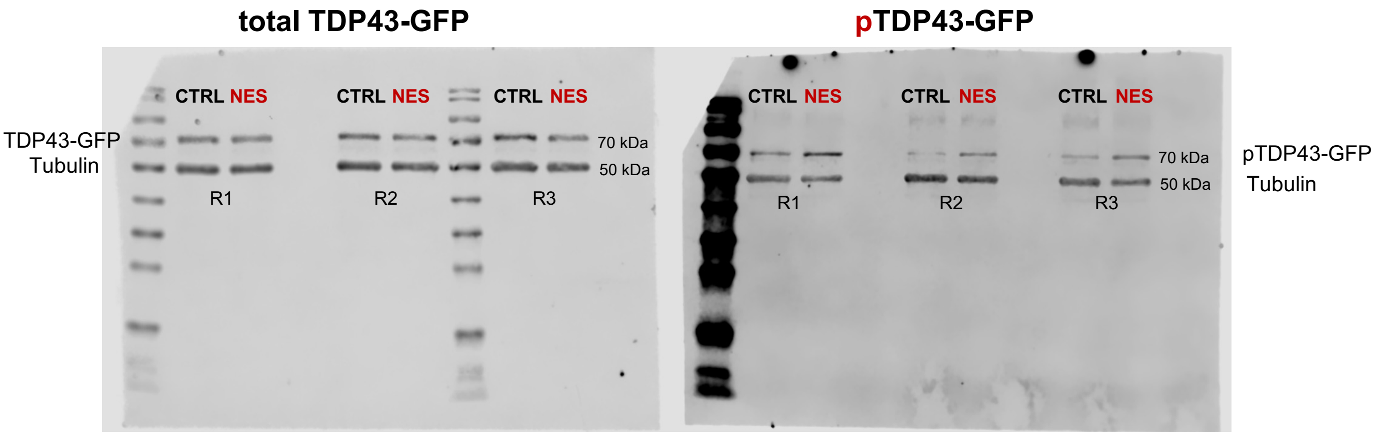

Figure 3 – Source Data 1. Uncropped Western Blots as seen in Figure 3D (and Figure 3-supplement 2) showing blots for total TDP43 and phosphorylated TDP43 (pTDP43) for control TDP43-GFP-CTRL and –NES lines. MN samples were collected at day 40, 20 days post-Dox addition. Alpha-tubulin (50 kDa) was used as a loading control.
